# Ontogeny and Trophic Factor Sensitivity of Gastrointestinal Projecting Vagal Sensory Cell Types

**DOI:** 10.1523/ENEURO.0511-22.2023

**Published:** 2023-04-19

**Authors:** Meaghan E. McCoy, Anna K. Kamitakahara

**Affiliations:** 1 The Saban Research Institute, Children's Hospital Los Angeles, Los Angeles, California 90027; 2 Keck School of Medicine, University of Southern California, Los Angeles, California 90033

**Keywords:** vagus, nodose ganglia, development, cell type, neurotrophic factor

## Abstract

Vagal sensory neurons (VSNs) located in the nodose ganglion provide information, such as stomach stretch or the presence of ingested nutrients, to the caudal medulla via specialized cell types expressing unique marker genes. Here, we leverage VSN marker genes identified in adult mice to determine when specialized vagal subtypes arise developmentally and the trophic factors that shape their growth. Experiments to screen for trophic factor sensitivity revealed that brain-derived neurotrophic factor (BDNF) and glial cell-derived neurotrophic factor (GDNF) robustly stimulate neurite outgrowth from VSNs *in vitro*. Perinatally, BDNF was expressed by neurons of the nodose ganglion itself, while GDNF was expressed by intestinal smooth muscle cells. Thus, BDNF may support VSNs locally, whereas GDNF may act as a target-derived trophic factor supporting the growth of processes at distal innervation sites in the gut. Consistent with this, expression of the GDNF receptor was enriched in VSN cell types that project to the gastrointestinal tract. Last, the mapping of genetic markers in the nodose ganglion demonstrates that defined vagal cell types begin to emerge as early as embryonic day 13, even as VSNs continue to grow to reach gastrointestinal targets. Despite the early onset of expression for some marker genes, the expression patterns of many cell type markers appear immature in prenatal life and mature considerably by the end of the first postnatal week. Together, the data support location-specific roles for BDNF and GDNF in stimulating VSN growth, and a prolonged perinatal timeline for VSN maturation in male and female mice.

## Significance Statement

Subpopulations of gastrointestinal-projecting vagal sensory neurons (VSNs) in the nodose ganglia convey satiety signals from the gut to the brain that regulate feeding behavior. Here, we examine gene expression in developing VSNs to better understand how they mature in perinatal life as they are exposed to ingestive stimuli. We demonstrate that thousands of genes are differentially expressed in the nodose ganglia of early postnatal mice compared with adults, indicating that VSNs continue to mature well after consumption of the first meal. The data further highlight the postnatal maturation of specific VSN subtypes and their sensitivity to trophic factors that stimulate growth and innervation of the gastrointestinal tract.

## Introduction

Abdominal-projecting vagal sensory neurons (VSNs) located in the nodose ganglion convey vital information from the gut to the brain that is used to regulate feeding behavior and coordinate descending motor control to the pancreas and gastrointestinal (GI) tract (Berthoud and Neuhuber, 2000; Schwartz, 2000; Travagli and Anselmi, 2016). GI-related sensory information is transmitted via specialized cell types that detect stomach or intestinal stretch, the presence of ingested nutrients, and/or the presence of microbial pathogens and inflammatory signals (Phillips and Powley, 1996; Wang and Powley, 2000; Powley and Phillips, 2004; Williams et al., 2016; Bellono et al., 2017; Beutler et al., 2017; Han et al., 2018; Kaelberer et al., 2018; Bai et al., 2019; Kupari et al., 2019; Ye et al., 2020). The sensory modalities and properties of individual VSN cell types have largely been defined in adult rodents, providing only a limited understanding of how and when specific vagal cell types arise and mature during embryonic and early postnatal life, and the factors that guide these developmental processes.

Studies that have examined vagal development provide a basic framework for understanding the trajectory of neurodevelopmental events from neurogenesis to functional circuits. A fused jugular–nodose complex forms from neural crest and placode cells (Douarin et al., 1986; Baker and Bronner-Fraser, 1997; Vermeiren et al., 2020). Only the ventrally positioned neurons of the nodose ganglion project to the GI tract. As early as embryonic day 10.5 (E10.5) in mice, VSNs begin to extend projections along the esophagus (Ratcliffe et al., 2011, 2006; Niu et al., 2020). Projections then grow rapidly to reach the stomach from E11.5 to E13.5 and reach the intestines between E14.5 and E16.5 (Ratcliffe et al., 2011, 2006; Niu et al., 2020). Intestinal morphology develops in concert with innervation. At E14.5, intestinal villi have yet to form, with few neuronal projections observed to enter the stromal space (Hao et al., 2020). Villi begin to emerge from E15.5 to E16.5, during which time neural projections enter the stroma to span the entire length of the villus (Hao et al., 2020).

While vagal projections extend the entire length of the GI tract prenatally, there is a lengthy period during the first few postnatal weeks when the connectivity matures (Smith, 2006; Murphy and Fox, 2007). For example, this has been shown in mechanosensitive VSN axons specialized to detect GI stretch that form intraganglionic laminar endings (IGLEs) and intramuscular arrays (IMAs). The innervation patterns of these neurons continue to mature morphologically throughout the first postnatal week in rodents (Murphy and Fox, 2007). At this time, gastric distention clearly inhibits feeding behavior in early postnatal rats, while the ability of ingested nutrients to inhibit feeding behavior does not arise until after the second postnatal week [approximately postnatal day 15 (P15) to P18; Phifer et al., 1986; Weller, 2000]. Together, the anatomic and behavioral data provide complementary temporal information on the developmental trajectory of VSN development.

The mechanisms guiding these critical developmental events remain only partially defined. Early neuronal survival and the growth of VSNs are known to depend on the support of brain-derived neurotrophic factor (BDNF) and glial-derived neurotrophic factor (GDNF; Ernfors et al., 1994; Moore et al., 1996; Niwa et al., 2002; Hellard et al., 2004). Furthermore, the density of IGLEs in the stomach are reduced following BDNF deletion, providing evidence that BDNF may support VSN types that project to the GI tract (Murphy and Fox, 2010). It is unknown whether other vagal subpopulations are similarly responsive, and experiments to address this are complicated by the fact that BDNF deletion is lethal.

Recent single-cell sequencing experiments have identified several unique VSN types, defined in adult mice (Bai et al., 2019; Kupari et al., 2019). These molecularly defined cell type markers may be useful to further identify which vagal subpopulations are affected by specific trophic factors as well as the developmental time points at which cell type-specific populations arise. Thus, the present study used bulk RNA sequencing and pathway analysis, combined with *in vitro* explant studies and *in situ* hybridization, to determine the molecular pathways and processes that underlie the development of VSNs. Our results reveal important relationships between trophic factors and specific vagal subpopulations and provide novel information on the timing of vagal cell type maturation.

## Materials and Methods

### Animals

Animal care and experimental procedures were performed in accordance with the Institutional Animal Care and Use Committee of The Saban Research Institute of Children’s Hospital Los Angeles. Mice were housed in the vivarium on a 13 h light/11 h dark cycle (lights on at 6:00 A.M., lights off at 7:00 P.M.) at 22°C with *ad libitum* access to a standard chow diet (PicoLab Rodent Diet 20; catalog #5053, LabDiet).

The C57BL/6J, *Phox2b^cre^*, and cre-dependent TdTomato reporter lines (*TdTomato*) were obtained from The Jackson Laboratory [C57BL/6J, strain 000664 (RRID:IMSR_JAX:000664); B6(Cg)-Tg(Phox2b-cre)3Jke/J, strain 016223 (RRID:IMSR_JAX:016223); and B6.Cg-Gt(ROSA)26Sortm14(CAG-tdTomato)Hze/J, stock 007914; Ai14 (RRID:IMSR_JAX:007914), respectively]. All lines were bred and maintained in The Saban Research Institute vivarium on a C57BL/6J background.

### RNA sequencing

Tissue from the nodose ganglion was dissected from male mouse pups on P7 and from male and female 12-week-old C57BL/6J mice. The dissection procedure was performed using a 1–2 cm incision made in the dermis overlying the larynx and esophagus. With the aid of a dissecting microscope, sterile forceps were used to gently retract the submaxillary glands away from the midline. The overlying stylohyoid and sternal muscles were removed, and the external carotid artery was pulled away from the vagus nerve to expose the underlying nodose ganglion. Sterile Dumont forceps were used to isolate and finely dissect the nodose ganglion away from the vagal trunk. While the nodose ganglion is fused together with the jugular ganglion, transection of the fused ganglia at the narrowing between the two facilitated enrichment of the sample by neurons of nodosal origin. Each sample consisted of the left and right nodose ganglia from a single mouse obtained from independent litters.

RNA was isolated from each sample using the RNeasy micro kit (catalog #74004, QIAGEN) as specified by the manufacturer. Total RNA quality was determined by measuring the RNA Integrity Number on an Agilent Bioanalyzer RNA Pico chip. Using 50 mg of RNA, transcriptome libraries were prepared and processed with a Next Ultra II Directional RNA Library Prep Kit (catalog #E7765, New England BioLabs) with single indexing following the manufacturer protocol. Library quality was determined using a Bioanalyzer DNA 1000 chip (Agilent). Libraries were sequenced using paired end (150 × 150 bp) chemistry on the HiSeq platform (Illumina; 350 M reads).

Reads were mapped to the University of California, Santa Cruz, mm10 transcript set using Bowtie2 version 2.1.0, and gene count was estimated using RSEM version 1.2.15. The trimmed mean of M values (TMM) method was used for gene count normalization to adjust for differences in sequencing depth and library size between samples, and differentially expressed genes were identified using the edgeR R program (Robinson and Oshlack, 2010). Genes exhibiting fold changes >1.5 with false discovery rate-adjusted *p*-values < 0.05 were considered differentially expressed.

Raw and processed data have been deposited in the GEO (Gene Expression Omnibus) and are available under accession number GSE227845.

### Ingenuity pathway analysis

Differentially expressed genes (DEGs) between P7 and 12-week-old male samples were further analyzed using Ingenuity Pathway Analysis software (Fall 2020 release, QIAGEN). To restrict the analysis to the highest confidence DEGs, cutoffs for average expression counts >5, expression *z* scores <2, fold changes >1.5, and adjusted *p*-values <0.05 were used. Using this list of analysis-ready molecules, statistically significant canonical pathways and upstream regulators were identified.

### Immunohistochemistry

Tissue for immunofluorescence staining was collected on E13 and E15, and from P0 and P7 mice. For embryonic tissue collection, timed pregnant breeding pairs were set, with the day of vaginal plug detection designated as E0. Sex was not determined for embryonic samples. Embryonic and P0 tissues were collected and immersed overnight in fixative (4% paraformaldehyde in 0.1 m PBS, pH 7.4). P7 mice were deeply anesthetized by intraperitoneal injection of ketamine/xylazine (100:10 mg/kg; Henry Schein Medical) and perfused transcardially with 0.9% saline, followed by fixative. Collected tissues were postfixed for 2 h, cryoprotected overnight in 20% sucrose in PBS, embedded in Tissue-Tek Optimal Cutting Temperature Compound, and frozen over liquid nitrogen vapors or powdered dry ice. From whole embryos or dissected nodose ganglion tissue, 20-μm-thick cryostat sections were collected in five series representing the entire width of the nodose ganglion. For whole embryos, sections were collected in the sagittal plane. For dissected nodose ganglion tissue, sections were collected longitudinally. Slides were stored at −20°C until processed.

For immunofluorescence labeling to detect cells undergoing cell death, slides were incubated for 1 h in blocking buffer containing 5% normal donkey serum (Jackson ImmunoResearch) and 0.3% Triton X-100 in PBS, then overnight in antibody solution containing 2% normal donkey serum and 0.3% Triton X-100 in PBS with primary rabbit anti-activated caspase 3 antibody (catalog #9661, Cell Signaling Technology). Sections were washed five times for 5 min in PBS, then incubated in secondary Alexa Fluor 594 AffiniPure F(ab')_2_ Fragment Donkey Anti‐Rabbit IgG (catalog #711–586-152, Jackson ImmunoResearch; RRID:AB_2340622). After several rinses in PBS, sections were counterstained with DAPI (catalog #D1306, Thermo Fisher Scientific), and a coverslip was applied using ProLong Gold Antifade Mountant (catalog #P36930, Thermo Fisher Scientific).

### Explant culture

From P0 *Phox2b^cre^;tdTomato* pups, both the left and right nodose ganglion were collected in Hibernate-A media (catalog #HA, BrainBits) containing B27 supplement (catalog #17504044, Thermo Fisher Scientific) and GlutaMAX (catalog #35050061, Thermo Fisher Scientific) and plated on polylysine-coated chamber slides in a droplet of growth factor reduced matrigel (catalog #354230, Corning). Explants were incubated with serum-free media containing Neurobasal media, B27 supplement, GlutaMAX, and penicillin/streptomycin. To each well, either vehicle (PBS) or candidate factors were added at the following concentrations: BDNF, 5 ng/ml (catalog #248-BDB-005, R&D Systems); GDNF, 5 ng/ml (catalog #RP-8602, Thermo Fisher Scientific); nerve growth factor (NGF), 50 ng/ml (catalog #256-GF-100, R&D Systems); leukemia inhibitory factor (LIF), 10 ng/ml (catalog #PHC9484, Thermo Fisher Scientific. The concentrations used are based on previously published work demonstrating the effects of neurite outgrowth in the nodose ganglion and in other neural tissues (Niwa et al., 2002; Schaller et al., 2017). Explant cultures were grown for 4 d in a standard 37°C incubator then imaged for analysis of neurite outgrowth.

### *In situ* hybridization

#### RNAscope

Paraformaldehyde fixed frozen samples from E13 and E15 whole mouse embryos were collected, and sections containing the nodose ganglion and gastrointestinal tissue were processed for multiplex fluorescent *in situ* hybridization (ACD RNAscope, Bio-Techne) using standard protocols specified by the manufacturer. Briefly, slides were baked for 1 h at 60°C, postfixed in 4% paraformaldehyde, and boiled in antigen retrieval reagent before hybridization with probes directed against mouse *Bdnf* or *Gdnf* mRNAs. Bound probes were detected using the RNAscope version 2 multiplex fluorescent detection kit and Opal 520 and 690 Dyes (Akoya Biosciences).

#### Dual HiPlex RNAscope and immunohistochemistry

Paraformaldehyde fixed frozen samples containing the right nodose ganglion were collected on E13, E15, P0, and P7 for processing for highly multiplexed mRNA labeling using the HiPlex RNAscope version 2 platform from Bio-Techne. As differences have been reported between the left and right nodose ganglion, all histologic analyses were performed solely on the right nodose ganglion for consistency (Han et al., 2018). Cryostat-prepared 20-μm-thick tissue sections were collected and mounted onto Superfrost Plus slides, baked at 60°C, and dehydrated in ethanol. Following antigen retrieval and protease treatments, sections were hybridized for 2 h against the following 12 mouse probes: T1-*Scn10a*, T2-*Cckar*, T3-*Htr3b*, T4-*Ntrk2*, T5-*Gfra1*, T6-*Vip*, T7-*Calca*, T8-*Gpr65*, T9-*Oxtr*, T10-*Slc17a6*, T11-*Glp1r*, and T12-*Sst*. Positive and negative control probe sets were run in parallel. Probe detection was performed in four rounds to detect T1–T3, T4–T6, T7–T9, and T10–T12 sequentially, accomplished with a confocal imaging step and fluorophore cleavage step between each round. A DAPI counterstain was also included in each round to serve as an anchor for later image registration.

Following the detection of all probes, tissue was processed for immunohistochemistry to detect the boundaries of neuronal cell bodies. Slides were incubated for 30 min in blocking buffer containing 10% normal donkey serum (Jackson ImmunoResearch) and 1% bovine serum albumin (BSA) in PBS, then overnight in antibody solution containing 1% BSA in PBS with primary mouse anti-HuC/D antibody (catalog #A-21271, Thermo Fisher Scientific; RID:AB_221448). Sections were washed two times for 5 min in PBS containing 0.1% Tween-20 (PBST), then incubated in secondary Alexa Fluor 594 AffiniPure F(ab')_2_ Fragment Donkey Anti‐Mouse IgG (Jackson ImmunoResearch catalog # 715–586-151, RRID:AB_2340858) antibody solution. After several rinses in PBST, sections were counterstained with DAPI (catalog #D1306, Thermo Fisher Scientific), and a coverslip was applied using ProLong Gold Antifade Mountant (catalog #P36930, Thermo Fisher Scientific). A final round of confocal imaging was then performed to capture HuC/D and DAPI labeling.

### Image acquisition

Laser-scanning confocal microscopes (STELLARIS, Leica; or LSM 710, Zeiss) equipped with 10×, 20×, 40× water-corrected, and 63× oil-corrected objectives were used to acquire fluorescence images. Confocal image stacks were collected through the *z*-axis at a frequency optimally determined by the imaging software based on the optics of the microscope and the wavelength of the fluorophores used for analysis. For all analyses, slides were coded so that the operator was blind to experimental group.

For analysis of activated Caspase^+^ and tdTomato^+^ neurons in the right nodose ganglion, every fifth consecutive section was imaged through the entire length of the ganglion using a 20× objective. For analysis of RNAscope labeling, the right nodose ganglion and intestinal tissue were identified and imaged using a 40× water-corrected objective. For analysis of HiPlex RNAscope labeling, two sections through the largest portion of the ganglion were captured in confocal image stacks using a 40× water-corrected objective. All images were collected on the STELLARIS confocal microscope (Leica), with the exception of the nodose ganglion explants, which required the use of a longer working distance lens that is only available in the Cellular Imaging Core at the Saban Research Institute on the LSM 710 confocal microscope (Zeiss). For explant images, the entire explant was imaged using a 10× objective and a *z*-step of 1 μm.

### Image analysis

For cell counts of activated Caspase^+^ and tdTomato^+^ neurons in the nodose ganglion, all image stacks for each animal were manually analyzed using the “Cell Counter” plugin within the FIJI/ImageJ software. The boundaries of the nodose ganglion and cell inclusion/exclusion criteria were defined before cell counts. The narrowing of tissue between the jugular and nodose ganglion served as a morphologic boundary for nodose ganglion cell counts. Only activated caspase colocalized with tdTomato was included in cell counts. This accounted for the vast majority of the caspase signal in the tissue (86%). Average profile diameter in each sample was measured and used to calculate estimated total cell counts in accordance with the formula of Abercrombie (1946).

Neurite outgrowth from nodose ganglion explants was quantified with the user blinded to treatment using FIJI/ImageJ software. Image stacks were projected through the *z*-axis, and signal was manually thresholded and binarized. Four neurite length measurements were made on each image in each cardinal direction from the edge of the explant to the tip of the neurite, for which values were averaged.

### Single-cell automated multiplex pipeline for RNA quantification and spatial mapping

Following dual HiPlex RNAscope, immunohistochemistry, and imaging procedures, as detailed above, confocal image stacks were analyzed using the single-cell automated multiplex pipeline for RNA (SCAMPR) quantification and spatial mapping (Ghoddousi et al., 2022). SCAMPR facilitates the analysis of highly multiplexed mRNA labeling at single-neuron resolution. Briefly, image stacks corresponding to each tissue section were projected across the *z*-axis and aligned using ACD image registration software (Bio-Techne), with DAPI being the common signal used to anchor all images together. Automated cell segmentation was performed using the Cellpose algorithm in Google Colaboratory based on HuC/D labeling to identify neuronal soma boundaries (Stringer et al., 2021). ROI boundaries outlining each neuronal cell body were imported into FIJI/ImageJ and manually corrected for any inappropriately assigned cell profiles. Individual images for each mRNA probe signal were then processed in ImageJ to subtract background (rolling ball radius = 1) and thresholded to facilitate the maximal detection of signal and removal of noise. Signal was binarized and the area fraction of the mRNA signal was measured for individual neuronal ROIs. Area fraction data were exported to a gene by cell matrix for further statistical analysis using GraphPad Prism and SCAMPR R scripts.

### Quantification and statistical analysis

Data were statistically analyzed and graphed using R or GraphPad Prism software (RRID: SCR_002798) and were expressed as mean ± SEM values. Each mouse is considered to be a sample (or each ganglion in the case of explant cultures), with sample sizes for each group and statistical analysis used included in each figure legend. A D’Agostino–Pearson normality test was used to determine whether parametric or nonparametric statistical analyses should be performed. For data following a normal distribution, an ordinary one-way ANOVA was used to compare means. For data that failed to pass the D’Agostino–Pearson normality test, a nonparametric Kruskal–Wallis test (correcting for multiple comparisons using Dunn’s test) was used to compare mean rank difference. A *p*-value < 0.05 was used for significance.

## Results

### Dynamic changes in gene expression in the developing nodose ganglion

**Figure 1.**
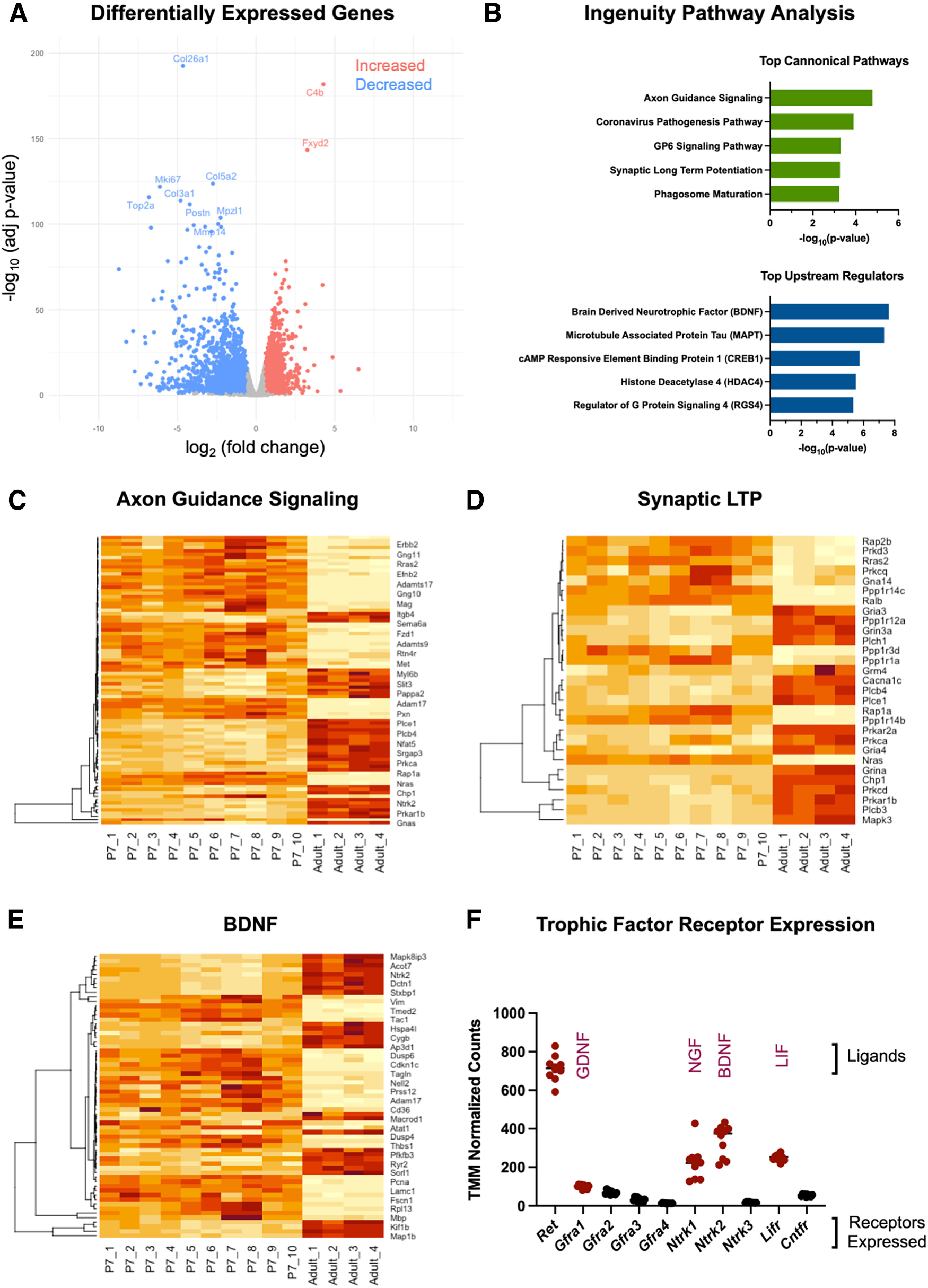
Transcriptomics analysis of nodose ganglion development reveals thousands of differentially expressed genes. ***A***, Volcano plot showing the 1453 genes that significantly increased (red; adjusted *p*-values < 0.05, fold changes > 1.5) and 1734 genes that significantly decreased (blue; adjusted *p*-values < 0.05, fold changes less than −1.5) in adult male nodose ganglion samples compared with P7 samples (*n* = 10 P7 samples; *n* = 4 adult samples). ***B***, Ingenuity pathway analysis of DEGs revealed the top canonical pathways and top upstream regulators in early postnatal versus adult samples. ***C***, Heatmap of DEGs related to axon guidance signaling. Warmer colors indicate higher gene expression. Individual samples are represented across columns. Specific genes are organized across rows grouped using hierarchical clustering. ***D***, Heatmap of DEGs related to synaptic LTP. ***E***, Heatmap of DEGs regulated by BDNF. ***F***, TMM-normalized counts of trophic factor receptors expressed in P7 nodose ganglion samples. Values highlighted in red were the top 5 highest expressed on P7 and were chosen for further testing in *in vitro* explant experiments.

To gain initial insight into how VSNs mature between early postnatal life and adulthood, RNA sequencing was performed comparing nodose ganglion samples from P7 and 12-week-old male mice. This analysis revealed 1453 genes that were significantly increased and 1734 genes that were significantly decreased in adults (Fig. 1*A*), representing >20% of the genes detected globally (3188 of 14,840 genes). The data suggest that there are dramatic transcript-level changes in VSNs from early postnatal life to adulthood. By comparison, when adult male and female samples were analyzed using the same cutoffs, only 11 genes were found to be differentially expressed, suggesting that little age-matched sexual dimorphism exists in the transcriptome of the mature nodose ganglion (refer to Extended Data Table 1-3 for full list).

10.1523/ENEURO.0511-22.2023.tab1-1 Table 1-1 Canonical pathways identified from RNA sequencing comparing nodose ganglia from P7 and 12-week-old mice. Differentially expressed genes (in “Molecules” field of the table) related to each Ingenuity canonical pathway are listed along with -log(*p*-values). Download Table 1-1, XLS file.

10.1523/ENEURO.0511-22.2023.tab1-2 Table 1-2 Upstream pathways identified from RNA sequencing comparing nodose ganglia from P7 and 12-week-old mice. Differentially expressed genes (in “Target molecules in dataset” field of the table) related to each upstream pathway are listed along with -log(*p*-values). Download Table 1-2, XLS file.

10.1523/ENEURO.0511-22.2023.tab1-3 Table 1-3 DEGs identified from RNA sequencing comparing nodose ganglia from male and female 12-week-old mice. Cutoffs of fold change >1.5, false discovery rate <0.05, and TMM-normalized counts >5 were used to identify high-confidence DEGs. Download Table 1-3, CSV file.

To further elucidate the biological implications of the large number of gene expression changes observed, QIAGEN Ingenuity Pathway Analysis was performed on the set of developmentally regulated DEGs. Among the top canonical pathways identified, axon guidance signaling and synaptic long-term potentiation (LTP; Fig. 1*B–D*) were identified, consistent with continued circuit growth, maturation, and synaptic strengthening between P7 and adulthood. Among the genes associated with synaptic long-term potentiation were many AMPA and NMDA receptor subunits as well as potassium and sodium transporters that modulate neuronal physiology and maturation (Extended Data Table 1-1). Another pathway identified, the coronavirus pathogenesis pathway, was heavily composed of ribosomal protein genes known to be important for viral replication, but in this context may relate more closely to their critical function in the increased translation that is required for neurite outgrowth. Similarly, the DEGs enriched within the GP6 signaling pathway were primarily composed of collagen-related and laminin-related genes that form the extracellular substrate that supports neurite outgrowth (refer to extended materials for full DEG and pathway lists). Among the top upstream regulators identified were BDNF, which has known roles in neuronal growth and survival, and microtubule-associated protein tau, which functions to stabilize axonal microtubules (Fig. 1*B*,*E*, Extended Data Table 1-2). Together, the sequencing data and pathway analysis demonstrate the substantial representation of genes involved in neurite growth, synaptic strengthening, and trophic factor signaling through BDNF.

BDNF is known to support the survival and growth of a subset of neurons in the nodose ganglion (Ernfors et al., 1994; Niwa et al., 2002; Hellard et al., 2004). This is consistent with our transcriptomics data suggesting that BDNF is a significant regulator of developmental gene expression on P7. Further analysis of the sequencing data confirmed high expression of the BDNF receptor, *Ntrk2*, at P7 along with several other neurotrophic factor receptors (NTFRs) with well established effects on nervous system development. Among the other most highly expressed NTFRs were *Ret* and *Gfra1*, which together bind GDNF; *Ntrk1*, the receptor for NGF; and *Lifr*, the receptor for LIF (Fig. 1*F*). Six other NTFRs exhibited limited expression. Given the prominent representation of DEGs involved in axon growth and guidance in early postnatal life, we sought to examine the relationship between the highest expressed trophic factor receptors in early postnatal samples and growth of VSNs.

### BDNF and GDNF support neurite outgrowth from the nodose ganglion

**Figure 2.**
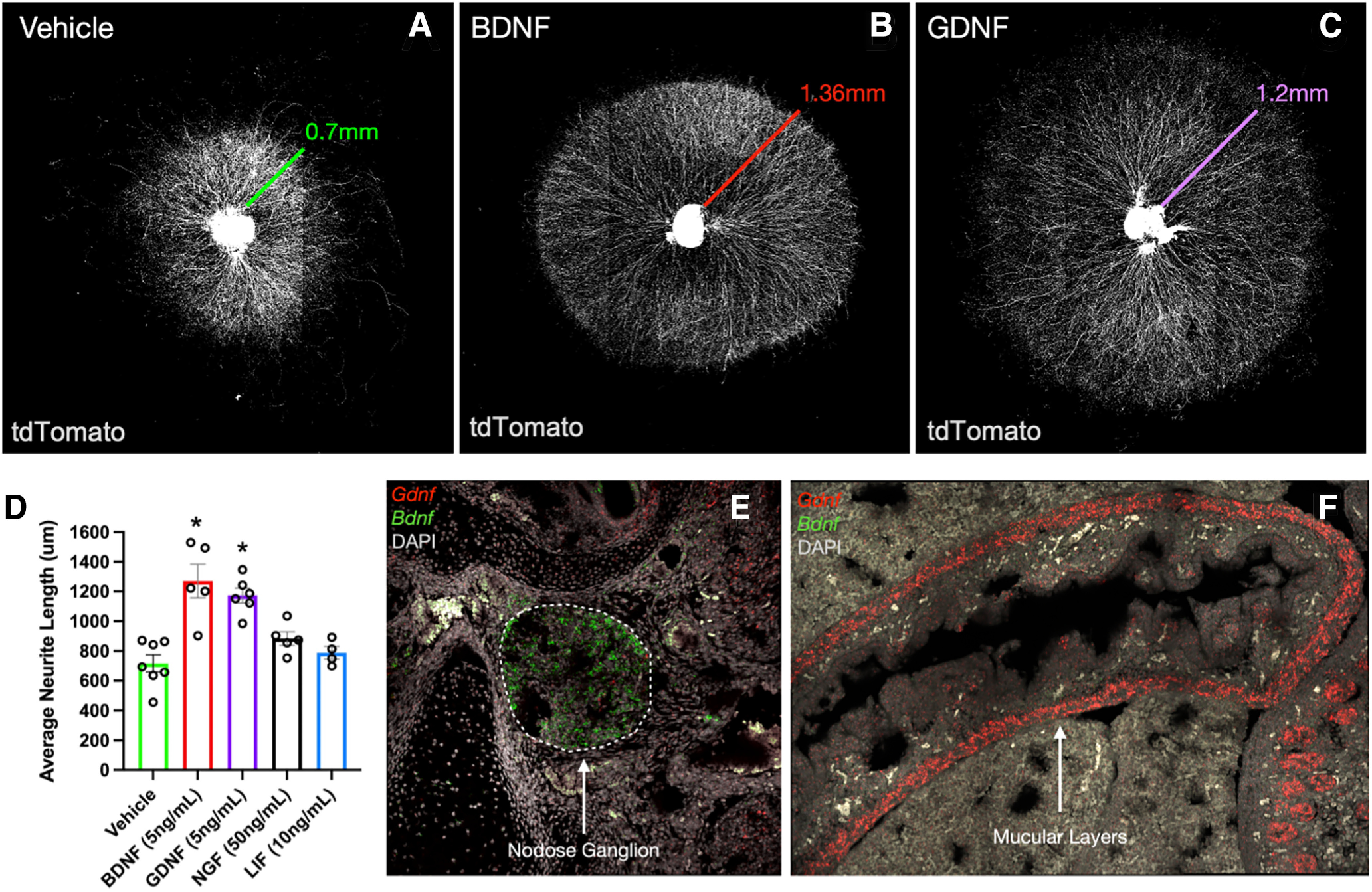
BDNF and GDNF stimulate neurite outgrowth from cultured nodose ganglion explants. ***A–C***, Representative images of nodose ganglion explants grown from *Phox2b^cre^;tdTom* mouse pups on P0 treated with vehicle (***A***), 5 ng/ml BDNF (***B***), or 5 ng/ml GDNF (***C***). Colored lines indicate average neurite lengths for each condition, as graphed in ***D***. To focus specifically on neurite outgrowth and to reduce the effects of neurotrophic factors on neuronal survival, ganglia were collected on P0 after the peak in apoptosis (Extended Data Fig. 2-1). ***D***, Quantification of average neurite length of nodose ganglion explants grown for 4 d with vehicle, BDNF, GDNF, NGF, or LIF. *p < 0.05 as analyzed by Kruskal–Wallis nonparametric ANOVA with Dunn’s correction for multiple comparisons. *n* = 6 vehicle, *n* = 5 BDNF, *n* = 5 GDNF, *n* = 3 NGF, *n* = 2 LIF. ***E***, ***F***, Representative images of multiplex *in situ* hybridization for *Bdnf* (green) and *Gdnf* (red), and DAPI counterstain (gray) in E15 mouse embryos. Dashed lines in ***E*** outline the boundaries of the right nodose ganglion. Arrow in ***F*** points to *Gdnf* transcript labeling in the muscular layers of the intestine.

Using an *in vitro* system of nodose ganglion explants, candidate neurotrophic factors were tested for their ability to stimulate neurite outgrowth. To focus specifically on neurite outgrowth and reduce the effects of neurotrophic factors on neuronal survival, ganglia were collected on P0 after the peak in apoptosis (Extended Data Fig. 2-1). Ganglia cultured with vehicle survived well and extended neurites growing radially in all directions from the explant (Fig. 2*A*). Compared with the vehicle-treated explants, ganglia cultured with either BDNF or GDNF exhibited robust increases in average neurite length (Fig. 2*A–D*). By contrast, ganglia cultured with NGF or LIF showed no difference in neurite length compared with vehicle-treated cultures (Fig. 2*D*). While differences in response properties and innervation patterns have been reported between the left and right nodose ganglion (Han et al., 2018), in the present experiments, no differences in trophic factor responsiveness between sides were observed.

10.1523/ENEURO.0511-22.2023.f2-1 Figure 2-1 Ontogeny of cell death in the nodose ganglion. ***A–D***, Representative images of the right nodose ganglion on E13, E15, P0, and P7 from *Phox2b^cre^;Tomato* mice. Immunohistochemistry was used to label the apoptotic marker, activated caspase 3 (magenta), together with endogenous tdTomato labeling (green) of all VSNs. ***E***, Quantification of the number of activated caspase-positive neurons per ganglion on E13, E15, P0, and P7 (*n* = 3–4 mice/group). *p < 0.05, **p < 0.01 comparing all groups by one-way ANOVA and Tukey’s correction for multiple comparisons. Download Figure 2-1, TIF file.

Given that BDNF and GDNF were both found to increase neurite outgrowth from developing nodose explants, we next determined their expression patterns during the perinatal period using multiplex fluorescent *in situ* hybridization (RNAscope). Interestingly, BDNF was highly expressed in the E15 nodose ganglion itself (Fig. 2*E*) in a pattern that overlapped with the neuronal marker HuC/D. Conversely, little GDNF was expressed in the nodose ganglion. Instead, GDNF was highly expressed in the muscular layers of the GI tract, and modestly expressed in stromal and epithelial compartments of the intestine (Fig. 2*F*). Together, the data show that while both BDNF and GDNF support nodose neurite outgrowth, BDNF may act locally within the nodose ganglion itself and GDNF may support target-derived growth of ganglion projections to subdiaphragmatic targets in the GI tract.

### GDNF receptor expression is enriched in subdiaphragmatic-projecting *Scn10a^+^* neurons

**Figure 3.**
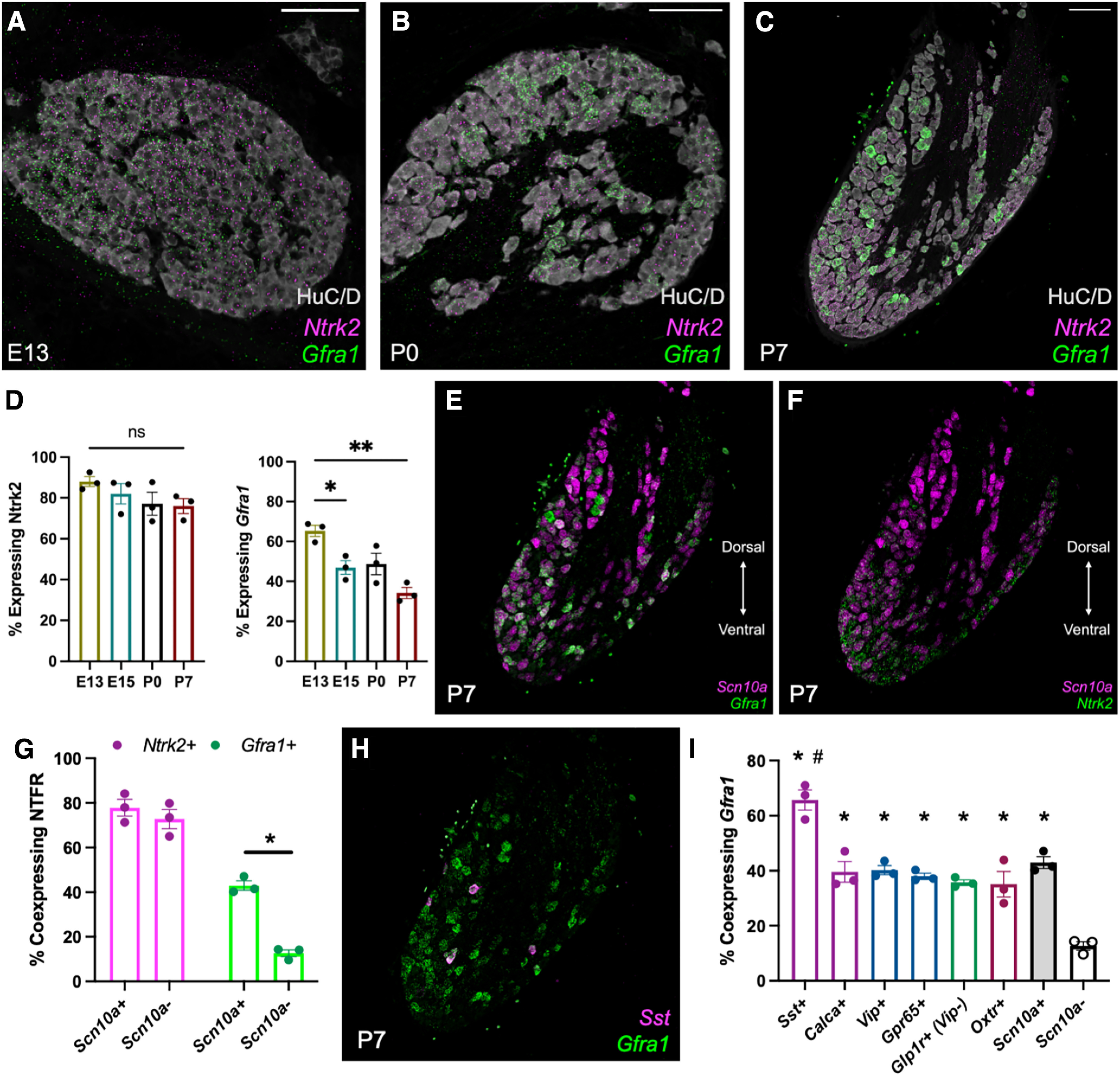
GDNF receptor expression is enriched in *Scn10a*^+^ vagal afferent cell types that project to the GI tract. ***A–C***, Representative images of the right nodose ganglia on E13 (***A***), P0 (***B***), and P7 (***C***) showing labeled *Gfra1* (green) and *Ntrk2* (magenta) transcripts, or the neuronal marker HuC/D (gray). ***D***, Quantification of the proportion of neurons expressing *Ntrk2* or *Gfra1* on E13, P0, and P7 (*n* = 3 mice/group). *p < 0.05, ***p < 0.001. ns, Not significant, as analyzed by one-way ANOVA and Tukey’s correction for multiple comparisons. ***E***, ***F***, Representative images of labeled *Scn10a* (magenta), *Gfra1* (green; ***E***), and *Ntrk2* (green; ***F***) transcripts in the right nodose ganglion of P7 mouse pups. ***G***, Quantification of the percentage of *Scn10a*^+^ and *Scn10a*^–^ that coexpress *Ntrk2* or *Gfra1* on P7 (*n* = 3 mice/group). Extended Data Figure 3-1, quantification on E13, E15, and P0. *p < 0.05 as analyzed by two-sided *t* test. Statistics for *Ntrk2* and *Gfra1* were performed separately, then graphed together. ***H***, Representative image of labeled *Sst* (magenta) and *Gfra1* (green) transcripts in the right nodose ganglia of a P7 mouse pup. ***I***, Quantification of the percentage of neurons expressing each cell type marker (*x*-axis) that coexpress *Gfra1* on P7. Values for *Scn10a*^+^ and *Scn10a*^–^ neurons also appear in ***G*** and are illustrated on the same graph for direct comparison (*n* = 3 mice/group). *p < 0.05 compared with *Scn10a*^–^ percentage coexpression by one-way ANOVA and Tukey’s correction for multiple comparisons. #*p* < 0.05 compared with *Scn10a*^+^ percentage of coexpression by one-way ANOVA and Tukey’s correction for multiple comparisons.

Responsiveness to BDNF and GDNF are dependent on expression of their respective neurotrophic factor receptors; *Ntrk2* for BDNF and *Gfra1* for GDNF. While the binding of GDNF to *Gfra1* also requires the expression of *Ret*, *Ret* dimerizes with other receptors such as *Gfra2*, *Gfra3*, and *Gfra4* to confer specificity of binding to other trophic factors (neurturin, artemin, and persephin, respectively) and is therefore not specific to GDNF (Airaksinen and Saarma, 2002). The enrichment of GDNF observed in the GI tract raises the question of whether the expression of the GDNF receptor *Gfra1* is also enriched in VSNs that project to the stomach and intestine. To this end, highly multiplexed *in situ* hybridization (HiPlex RNAscope) was used to label 12 independent RNA transcripts within the same tissue section. This facilitated the labeling of several cell type-specific VSN markers with *Ntrk2* or *Gfra1*. Tissue samples were collected from mice on E13, E15, P0, and P7 to examine trophic factor receptor expression as nodose sensory neurons grow and reach GI targets, and as they mature postnatally. Analysis across these ages demonstrated that *Ntrk2* is expressed in the majority of VSNs at all prenatal and postnatal ages examined (Fig. 3*A–D*). By contrast, *Gfra1* was expressed by a significantly higher fraction of nodose ganglion neurons at E13 than at E15 or P7. While the proportion of neurons expressing *Gfra1* declined, expression in the remaining Gfra1^+^ neurons was at high levels (Fig. 3*A–D*).

10.1523/ENEURO.0511-22.2023.f3-1 Figure 3-1 Neurotrophic factor receptor expression in *Scn10a*^+^ and *Scn10a*^–^ neurons across development. The percentage of neurons coexpressing either *Ntrk2* or *Gfra1* was measured on E13, E15, and P0 (*n* = 3 mice/group). *p < 0.05 as analyzed by two-sided *t*-test at each age. Statistics for *Ntrk2* and *Gfra1* were performed separately, then graphed together. Download Figure 3-1, TIF file.

To determine the subpopulation of neurons expressing *Gfra1* or *Ntrk2*, each NTFR was examined for colocalization with specific vagal cell type markers at P7. Anatomical and single-cell sequencing studies have demonstrated that *Scn10a* (also known as Nav1.8) is primarily expressed by VSNs that project below the diaphragm (∼90%), with a minor portion of afferents projecting to pulmonary targets (Bai et al., 2019; Kupari et al., 2019). At P7, *Ntrk2* was expressed by a similar proportion (∼75%) of *Scn10a*^+^ and *Scn10a*^–^ neurons (Fig. 3*F*,*G*). By contrast, only 12% of *Scn10a*^–^ neurons expressed *Gfra1*, while 43% of *Scn10a*^+^ neurons coexpressed *Gfra1*, demonstrating a significant enrichment of *Gfra1* in a neuronal population that projects nearly selectively to the GI tract (Gautron et al., 2011; Bai et al., 2019; Kupari et al., 2019; Fig. 3*E*,*G*). A distinct enrichment of *Gfra1*^+^ neurons was apparent in the ventral portion of the fused nodose–jugular ganglion, corresponding to expression in the ventral nodosal compartment. Together, the specific expression patterns are consistent with a role for target-derived GDNF expressed by the developing GI tract in supporting subdiaphragmatic vagal growth.

Subdiaphragmatic *Scna10a*^+^ vagal cell types can further be subtyped by the expression of other marker genes. Stomach mucosal-projecting neurons express either *Sst* or *Calca*, stomach IGLEs express *Glp1r* but not *Vip*, intestinal mucosal-projecting neurons express either *Gpr65* or *Vip*, and intestinal IGLEs express *Oxtr* (see Fig. 5*B*; Bai et al., 2019). Using HiPlex RNAscope, each of these cell types were examined for coexpression of *Gfra1* at P7. Compared with *Scn10a*^–^ neurons, all GI-projecting populations (*Sst*^+^, *Calca*^+^, *Vip*^+^, *Gpr65*^+^, *Glp1r*^+^/*Vip*^–^, and *Oxtr*^+^) exhibited a significant enrichment of *Gfra1* expression (Fig. 3*I*), with *Sst*^+^ stomach mucosal neurons exhibiting the highest enrichment of *Gfra1* compared with other *Scn10a*^+^ neuronal subtypes (Fig. 3*H*,*I*).

Potential *Ntrk2* and *Gfra1* enrichment in select subpopulations of nodose ganglion neurons was also analyzed in embryonic samples. *Scn10a* expression was first detected at E13, a day before descriptions in *Scn10a-Cre* mice where robust Cre-dependent X-gal staining was detected at E14 (Stirling et al., 2005). At E13, a similar proportion of *Scn10a*^+^ and *Scn10a*^–^ neurons coexpressed *Gfra1* (∼65%; Extended Data Fig. 3-1). By E15, coexpression of *Gfra1* was significantly decreased in *Scn10a*^–^ neurons (52.8% of *Scn10a*^+^, 31.1% of *Scn10a*^–^). This difference was sustained at P0 (57.9% of *Scn10a*^+^, 31.4% of *Scn10a*^–^) and P7 (43.0% of *Scn10a*^+^, 12.6% of *Scn10a*^–^) by nodose ganglia. A significant, but transient, decrease in the proportion of *Scn10a*^–^ neurons expressing *Ntrk2* also was observed at E15, but was not sustained in P0 or P7 nodose ganglion. While the analysis of coexpression within *Scn10a*^+^ neurons was possible at embryonic time points, the coexpression of NTFR in specific subpopulations of GI-projecting neurons was not possible because of the low expression of most cell type marker genes. Instead, expression patterns were leveraged to examine the maturation of cell type marker expression.

### Nodose cell types are readily resolved by early postnatal life

**Figure 4.**
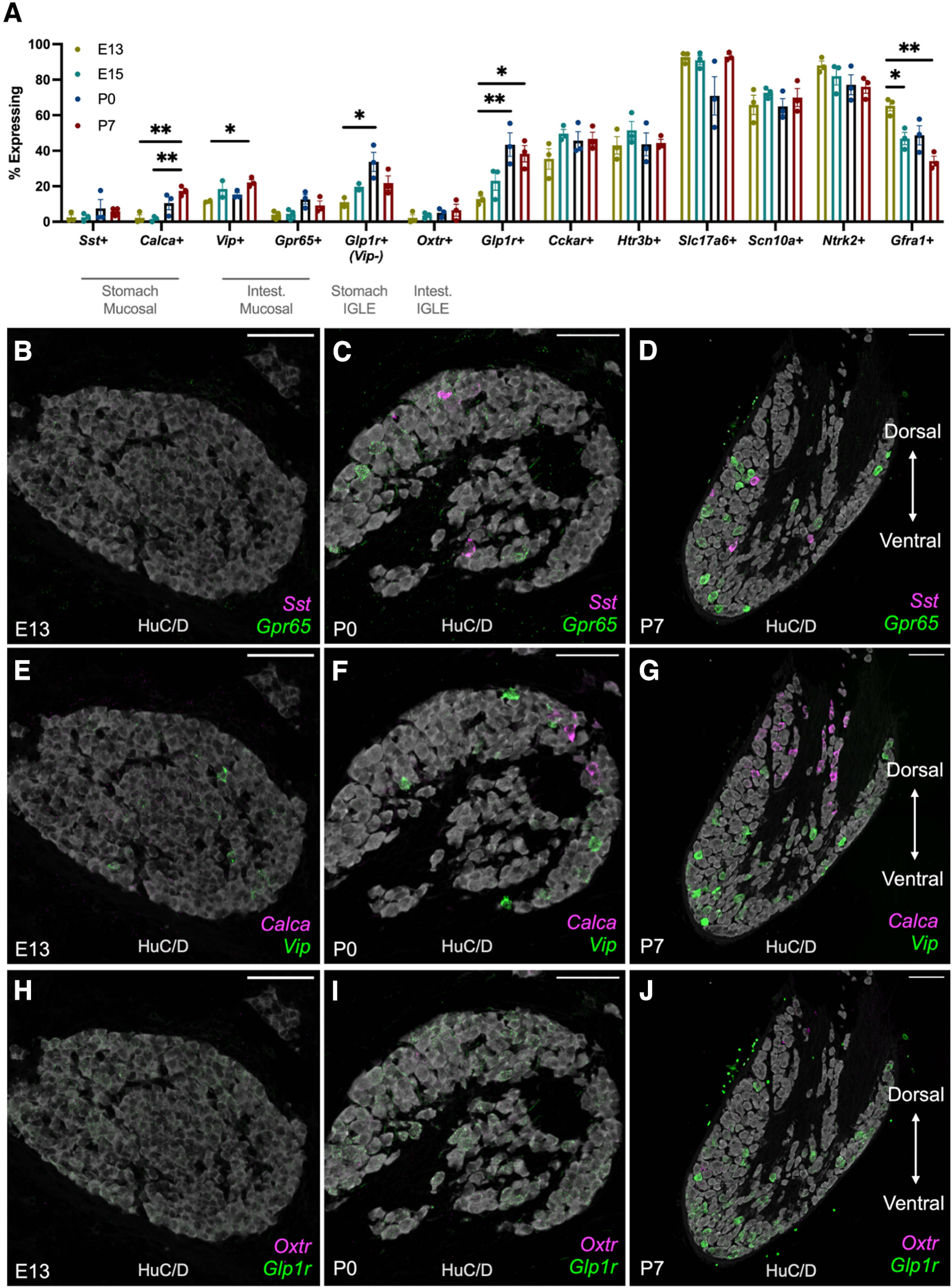
Early prenatal expression of vagal afferent cell type markers becomes progressively more mature by P7. ***A***, Quantification of all 12 transcripts measured using HiPlex RNAscope and the SCAMPR for analysis at single-neuron resolution. The mRNAs that were probed are indicated on the *x*-axis. The percentage of neurons expressing each transcript is indicated on the *y*-axis. Each point represents percentages calculated from a single mouse, in which expression was quantified from several hundred neurons in the right nodose ganglion (*n* = 3 mice/group; 324–795 neurons measured per sample). *p < 0.05 comparing all ages for each transcript by one-way ANOVA and Tukey’s correction for multiple comparisons. ***B–D***, Representative images of the right nodose ganglion on E13, P0, and P7 labeled for *Sst* (magenta) and *Gpr65* (green) transcripts and the neuronal marker HuC/D. Scale bar, 100 μm. ***E–G***, Representative images of the right nodose ganglion on E13, P0, and P7 labeled for *Calca* (magenta) and *Vip* (green) transcripts and HuC/D. Scale bar, 100 μm. ***H–J***, Representative images of the right nodose ganglion on E13, P0, and P7 labeled for *Oxtr* (magenta) and *Glp1r* (green) transcripts and HuC/D. Scale bar, 100 μm. For representative images of *Htr3b* and *Cckar* transcripts refer to Extended Data Figure 4-1.

Nodose ganglion cell type identity and function have primarily been examined in adult rodents (Gautron et al., 2011; Egerod et al., 2018; Bai et al., 2019; Kupari et al., 2019). As such, there are limited data available that describe the development of VSN transcriptomic diversity and cell type identity. To establish a timeline for VSN cell type-specific marker expression, HiPlex RNAscope and the SCAMPR analysis were used. On E13 and E15, little to no signal was concentrated within specific cell bodies for *Sst*, *Calca*, *Gpr65*, or *Oxtr* transcripts [Fig. 4*A*,*B*,*E*,*H*, representative E15 ganglia images not shown (but are quantified in Fig. 4*A*)]. Conversely, a dense *Vip* signal was observed in a small number of cell bodies at E13 (Fig. 4*E–G*). The proportion of *Vip*^+^ neurons increased through E15 and P7 (Fig. 4*A*,*E–G*). Additionally, neurons expressing the serotonin receptor *Htr3b* also exhibited high expression in a subpopulation of neurons that was maintained by a constant proportion of neurons from E13 to P7 (Fig. 4*A*, Extended Data Fig. 4-1). The expression patterns of *Vip* and *Htr3b* in specific neuronal subpopulations at E13 suggest that, while still immature, specific cell types begin to emerge just a few days after the ganglion forms. By P7, concentrated labeling for all cell type markers were localized to specific single-neuron profiles (Fig. 4*D*,*G*,*J*, Extended Data Fig. 4-1).

10.1523/ENEURO.0511-22.2023.f4-1 Figure 4-1 Early specification of subpopulations of vagal neurons expressing *Htr3b*. ***A–C***, Representative images of the right nodose ganglion on E13, P0, and P7 labeled for *Htr3b* (magenta) and *Cckar* (green) transcripts and HuC/D. Scale bar, 100 μm. Download Figure 4-1, TIF file.

A dorsal–ventral-specific labeling pattern was observed for several transcripts at P7. *Sst*, *Gpr65*, *Vip*, and *Glp1r* were primarily localized to the ventral portion of the nodose–jugular ganglion complex (Fig. 4*D*,*G*,*J*). Conversely, *Calca*^+^ neurons were localized to the dorsal portion of the ganglion (Fig. 4*G*). While *Calca* is expressed by ∼10% of gastric-projecting nodose neurons, it is also expressed by *Prdm12*^+^ neurons of the jugular ganglion, which are located in the dorsal-most portions of the fused jugular–nodose ganglion (Bai et al., 2019; Kupari et al., 2019).

**Figure 5.**
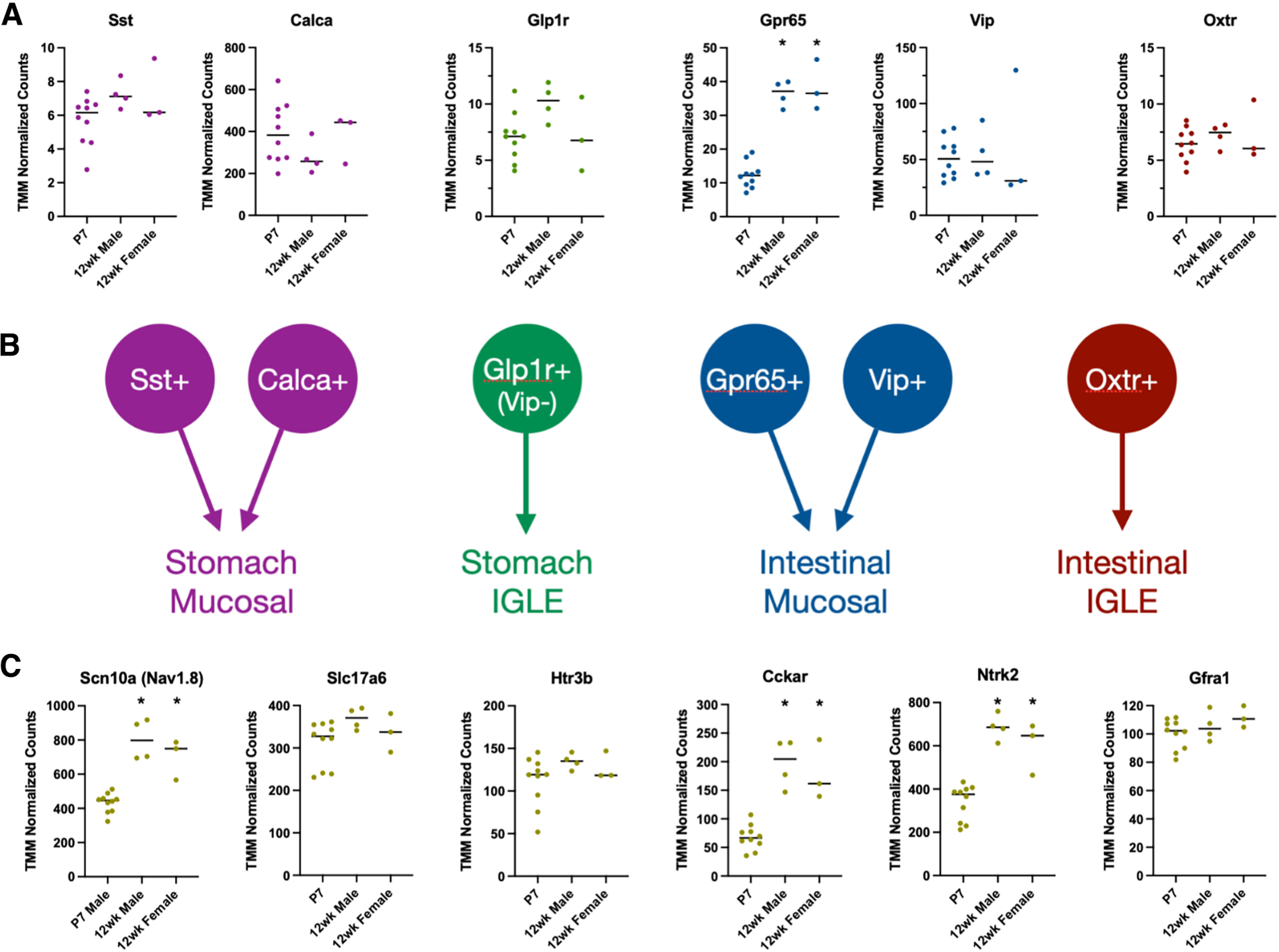
Expression of some vagal afferent cell type markers continues to increase after the first postnatal week. ***A***, TMM normalized counts of vagal afferent cell type markers expressed in P7 male and adult male and female nodose ganglion samples (*n* = 10 P7 male samples, *n* = 4 adult male samples, *n* = 3 adult female samples). ***B***, Schematic depicting each cell type marker and its respective subtype definition as projecting to the stomach versus intestine, and as an IGLE versus mucosal afferent neuron type. ***C***, TMM normalized counts of other notable cell-defining markers expressed in P7 male and adult male and female nodose ganglion samples. The markers graphed are the same ones probed in the HiPlex RNAscope experiments described in Figure 4 (*n* = 10 P7 male samples, *n* = 4 adult male samples, *n* = 3 adult female samples). *p < 0.05 comparing all groups by one-way ANOVA and Tukey’s correction for multiple comparisons.

Examination of RNA sequencing data were further used to compare changes in cell type marker gene expression between P7 male and adult male and female mice. No statistically significant differences in cell type marker expression were observed by comparing adult male and female nodose ganglion (Fig. 5*A*). Compared with adult ganglia, there was no change in expression for *Sst*, *Calca*, *Glp1r*, *Vip*, or *Oxtr*, suggesting that by P7 mature patterns of neuronal subtype expression have been reached for these genes in nodose neurons (Fig. 5*A*). By contrast, *Gpr65*, a marker of a subtype of intestinal macronutrient-responsive mucosal neurons, increased more than threefold in adult male and female nodose ganglion, suggesting that this population continues to mature after P7 (Fig. 5*A*; Williams et al., 2016). *Scn10a*, *Cckar*, and *Ntrk2* were also increased in adults (Fig. 5*C*). Nav1.8 (*Scn10a*) is a voltage-gated sodium channel that modulates vagal electrophysiological properties, while CCK A receptors (*Cckar*) participate in vagally mediated satiety signaling and meal termination (Gibbs et al., 1973; Raybould and Tache, 1988; Moran, 2000). The expression data for *Gpr65*, *Scn10a*, and *Cckar* suggest that the response properties of GI-projecting vagal neurons continue to mature in early postnatal life as they are exposed to feeding-related stimuli.

## Discussion

Cell type definitions established in adults through both genomic and functional studies have been instrumental to understanding adult vagal sensory communication and physiological regulation of ingestive behavior. The present study leverages these cell type definitions to further understand the timing of when vagal neuronal subtypes emerge developmentally and, moreover, their dependence on specific trophic influences. Here, we demonstrate that both BDNF and GDNF can stimulate *in vitro* neurite outgrowth from VSNs. Mapping of neurotrophic factor expression patterns developmentally indicates that while BDNF may act locally to support growth from the nodose ganglion itself, GDNF may act as a target-derived growth factor within the muscular layers of the intestine. Consistent with the latter, the GDNF receptor *Gfra1* is enriched preferentially in *Scn10a*^+^ neurons that primarily project below the diaphragm to GI sites, and in the vagal neuronal subtypes known to project to the GI tract. Last, highly multiplexed *in situ* hybridization data demonstrate that discrete cell type marker expression in specific subpopulations of nodose neurons can be detected as early as E13 for transcripts such as *Vip* and *Htr3b*, which is suggestive of specification of a subset of neuronal subtypes by this age. Our analysis did reveal, however, that the patterns of cell type marker expression are largely immature prenatally and further develop into discretely labeled subpopulations by the end of the first postnatal week.

### Transcriptomic data indicate large differences in gene expression in early postnatal life compared with adults

Transcriptomics analyses revealed that 20% of genes in the nodose ganglion are differentially expressed between P7 and adults. Heavily represented among these DEGs were transcripts involved in axon growth and guidance. This was somewhat surprising given past descriptions suggesting that on P7 vagal sensory circuit morphology in the GI tract is similar to patterns observed in adults (Murphy and Fox, 2007). Similarly, central projections of vagal axons to terminal regions in the nucleus of the solitary tract have been described as mature as early as P2 in rats (Rinaman and Levitt, 1993). Despite the relatively mature pattern of innervation observed in early postnatal life morphologically, smaller-scale increases in neurite outgrowth and in refinement at synapses may be at play. This is further supported by the enrichment of DEGs associated with synaptic LTP. It is clear from seminal behavioral studies that the ability for nutrient-related postingestive feedback to modulate feeding does not arise until after the second postnatal week (Phifer et al., 1986; Weller et al., 2000; Smith, 2006; Weller, 2006, 2000). Therefore, it is possible that some of the expression changes identified in the present study may relate to ongoing maturation and synaptogenesis in circuits required for nutrient detection.

In addition to the substantial number of DEGs identified through sequencing, there remain many transcripts that were not differentially expressed. These include an estimated 25% of genes that are constitutively expressed housekeeping genes found in all mouse tissues (Li et al., 2017). Other notable genes not differentially expressed in the sequencing experiment include many of the cell type marker genes (e.g., *Calca, Vip, Glp1r*). Instead, the expression of *Calca, Vip*, and *Glp1r* was found to significantly increase between E13 and P7, demonstrating the importance of timing in capturing specific gene expression changes. Separate, but partially overlapping changes in expression are likely in the perinatal developmental period examined by *in situ* hybridization (E13 to P7) compared with the postnatal time points sequenced (P7 to adult).

### Neurotrophic factor and receptor expression patterns and effects on VSN outgrowth

Neurotrophic factors are highly pleiotropic, having many biological functions ranging from neuronal proliferation, survival, differentiation, and synaptogenesis. Of these functions, our study and others together demonstrate that both BDNF and GDNF support VSN survival and neurite growth. *In situ* hybridization experiments demonstrate that while BDNF is expressed by neurons of the nodose ganglion, GDNF is expressed by distal targets in the GI tract. Together, these data suggest that BDNF acts as a locally derived signal at the level of the nodose ganglion itself, while GDNF works instead as a target-derived signal to support vagal neuron growth and survival. This hypothesis is additionally supported by a limited number of pilot *in vitro* experiments we performed where vagal neuronal survival was low in vehicle-treated dissociated cultures, in which any produced growth factors would be diluted in the culture media (data not shown). In contrast, vagal neuronal survival is high in explant cultures in which the cells are packed tightly together, suggesting that a factor produced within the ganglia acts locally to positively impact neuronal survival. Exogenous BDNF enhances explant outgrowth to a greater extent than vehicle alone, clearly demonstrating its importance in vagal sensory neurite growth. The exact subcellular site where BDNF is released remains to be determined as release from the cell body versus from centrally or peripherally projecting processes could have discrete functional consequences.

Experiments published by others demonstrate that gastric innervation is reduced in BDNF^−/−^ mice at P0 but recovers by P3 in the small number of mutant mice surviving to this age (Murphy and Fox, 2010). This is consistent with our data, suggesting that GDNF provides local trophic support in the GI tract and may compensate for the reductions in innervation in BDNF^−/−^ mice. Paradoxically, deletion of BDNF from the intestinal musculature results in an increase in vagal intraganglionic laminar ending density (Biddinger and Fox, 2014). Thus, target-derived BDNF can additionally affect VSN growth and maturation, despite its comparatively low expression at the prenatal ages assessed in the current study.

At all developmental ages tested, the BDNF receptor (*Ntrk2*) was expressed by the vast majority of neurons (78–88%), which is consistent with a broad impact on survival for most vagal neurons. By comparison, the pattern of expression of *Gfra1* was such that while it was expressed more widely but at lower levels in prenatal life, it became more highly expressed in a smaller subset of only 34% of neurons by P7. No experiments were performed to test the synergistic effects of both BDNF and GDNF together in the *in vitro* explant culture model. However, our *in situ* hybridization data demonstrate that ∼30% of all VSNs coexpress both *Ntrk2* and *Gfra1*, suggesting that synergistic effects are possible in this subset.

The data demonstrate that *Gfra1* is enriched in *Scn10a*^+^ populations compared with *Scn10a*^–^ populations and was similarly enriched among several functionally defined cell types, particularly in *Sst*^+^ neurons. Single-cell sequencing studies performed in adults suggest that *Gfra1* is prevalent in *Gpr65*^+^ populations that are mucosal-projecting, nutrient-sensing neurons (Williams et al., 2016; Kupari et al., 2019). Our results indicate no greater enrichment in *Gpr65*^+^ neurons compared with any other GI-projecting population; however, it is noteworthy that the sequencing data in the present study indicate the occurrence of a more than threefold increase in *Gpr65* expression between P7 and adulthood. It is possible that that this enrichment of *Gfra1* in *Gpr65*^+^ neurons occurs later in development after the first postnatal week.

### Timing of cell type marker expression

Initial expression of specific marker genes is a clear indication that neuronal type specification has been initiated. Our multiplex *in situ* hybridization data demonstrate that as early as E13, transcripts for *Vip* and *Htr3b* are already expressed within specific subpopulations of neurons. Additionally, we also found that the expression patterns of neuronal subtype-specific genes continue to mature considerably through postnatal life. In adult rodents, *Vip*^+^ neurons project exclusively to the mucosal compartment of the small intestine (Bai et al., 2019). Developmental studies demonstrate that vagal sensory axons first reach the intestine between E12 and E14 and begin to innervate the mucosal compartment between E14 and E16 (Ratcliffe et al., 2006; Hao et al., 2020; Niu et al., 2020). The timing of these developmental events together with the marker expression data from the current study demonstrate that defined *Vip*^+^ subpopulations arise either before or concurrent with reaching their terminal innervation targets.

In contrast to the data for *Vip*, most of the other marker genes did not exhibit restricted cell type-specific expression patterns until later time points. It is an important distinction to note that the appearance of the cell type markers used in the present study is only one measure of neuronal maturation. Some genetic markers may be expressed either earlier or later than other markers in neurons that are at comparable developmental states. For example, cell type may already be specified by E13 in stomach mucosal neurons despite not expressing high levels of *Sst* or *Calca*. Additional experiments will be required to more precisely define when these cell fate decisions occur for all vagal subpopulations. However, the *Vip* and *Htr3b* data along with the caspase-3 results in the current study suggest that some cell type specification already has occurred by E13 even as other developmental processes proceed, such as continued neurite outgrowth and apoptosis. Other published studies indicate that by P0, there are morphologically distinct projections within the GI tract characteristic of IGLEs, IMAs, and mucosal endings, suggesting that some vagal sensory cell types are clearly defined by birth (Murphy and Fox, 2007). Our data confirm and extend this timeline earlier, suggesting that defined cell types emerge prenatally.

In addition to the early onset of cell type-specific expression observed for some marker genes, prolonged maturation in expression was also noted for other marker genes in the bulk RNA sequencing dataset. Notably *Cckar* and *Gpr65* significantly increased in expression between P7 and 12 weeks. Both of these genes are important for nutrient-detecting vagal pathways. CCKA receptors detect CCK released from intestinal enteroendocrine cells in response to nutrient ingestion, and *Gpr65*^+^ neurons respond electrophysiologically to nutrient ingestion (Williams et al., 2016; Kaelberer et al., 2018). The finding that their expression increases more than threefold after P7 is consistent with other functional studies that have demonstrated that the ability of ingested nutrients to modulate feeding behavior develops after the second postnatal week (Phifer et al., 1986; Weller, 2000).

Together, our analyses demonstrate that VSN expression continues to change and mature postnatally in mice. While comparative indices of vagal sensory maturation are poorly defined in humans, the maturation of vagal motor tone has been shown to extend well over the first 3 years of life (Wagner et al., 2021). This continued postnatal maturation of functional properties raises the possibility that the early life nutritional environment, during the period of neuronal subtype maturation, could serve as a modulator of developmental trajectories and outcomes in the adult. The data provide potential biomarkers for vagal maturation in infants and a greater context for understanding therapies that target vagal function in pediatric populations, such as the vagus nerve stimulation used to manage epilepsy. Future studies will follow the present developmental findings to further parse key developmental events and to determine mechanisms that underlie vagal circuit maturation and function.

## Synthesis

Reviewing Editor: Masha Prager-Khoutorsky, McGill University Decisions are customarily a result of the Reviewing Editor and the peer reviewers coming together and discussing their recommendations until a consensus is reached. When revisions are invited, a fact-based synthesis statement explaining their decision and outlining what is needed to prepare a revision will be listed below. The following reviewer(s) agreed to reveal their identity: Laurent Gautron.

below

Synthesis of Reviews:

Reviewers acknowledged that this is an important study elucidating novel factors associated with the vagus nerve nodose ganglion.

There are several topics that require authors attention and should be addressed to make the manuscript suitable for publication.

1. It seems surprising that, considering the many profound changes accompanying the first weeks of postnatal life (weaning, gut permeability changes, immune acquisition), the authors did not observe more dramatic changes in the expression profiles of vagal afferent neurons. Please include some discussion on this.

2. In the discussion, please provide further discussion on the relevance of the findings in regards to clinical or other outcomes. This should also be briefly added to the abstract.

3. Neurotrophic factors can have unpesepcific roles. It should be expanded on the relevance of the findings on the differences in BDNF versus GDNF at the level of functions. Are there any functional studies of these markers? If so they should be cited.

4. Please provide detailed description of the procedure for dissecting the nodose as that is a key procedure in the methods.

5. In Extended figure 2-1, the caspase staining is hard to see. Could the authors possibly provide images a little less blurry and/or at higher magnification.

6. It is said that 11 genes were different between males and females? It would be interesting to include these genes in the manuscript.

7. Have the authors tried to culture vagal afferent neurons in the presence of both BDNF and GDNF? Would you expect a synergic effect?

8. Overall, the literature is accurately cited and the list of references comprehensive. The reviewer suggests citing PMID: 15621361 because they mention that Scn10a expression begins at in E14 in the mouse.

9. It is unclear what “TMM” stands for, and what the “TMM method” consists of?
