# Supplementary figures and images for: Ontogeny and Trophic Factor Sensitivity of Gastrointestinal Projecting Vagal Sensory Cell Types
Source: eNeuro. 2023 Apr 19;10(4):ENEURO.0511-22.2023. doi: 10.1523/ENEURO.0511-22.2023 (PMC10124152; doi:10.1523/ENEURO.0511-22.2023)

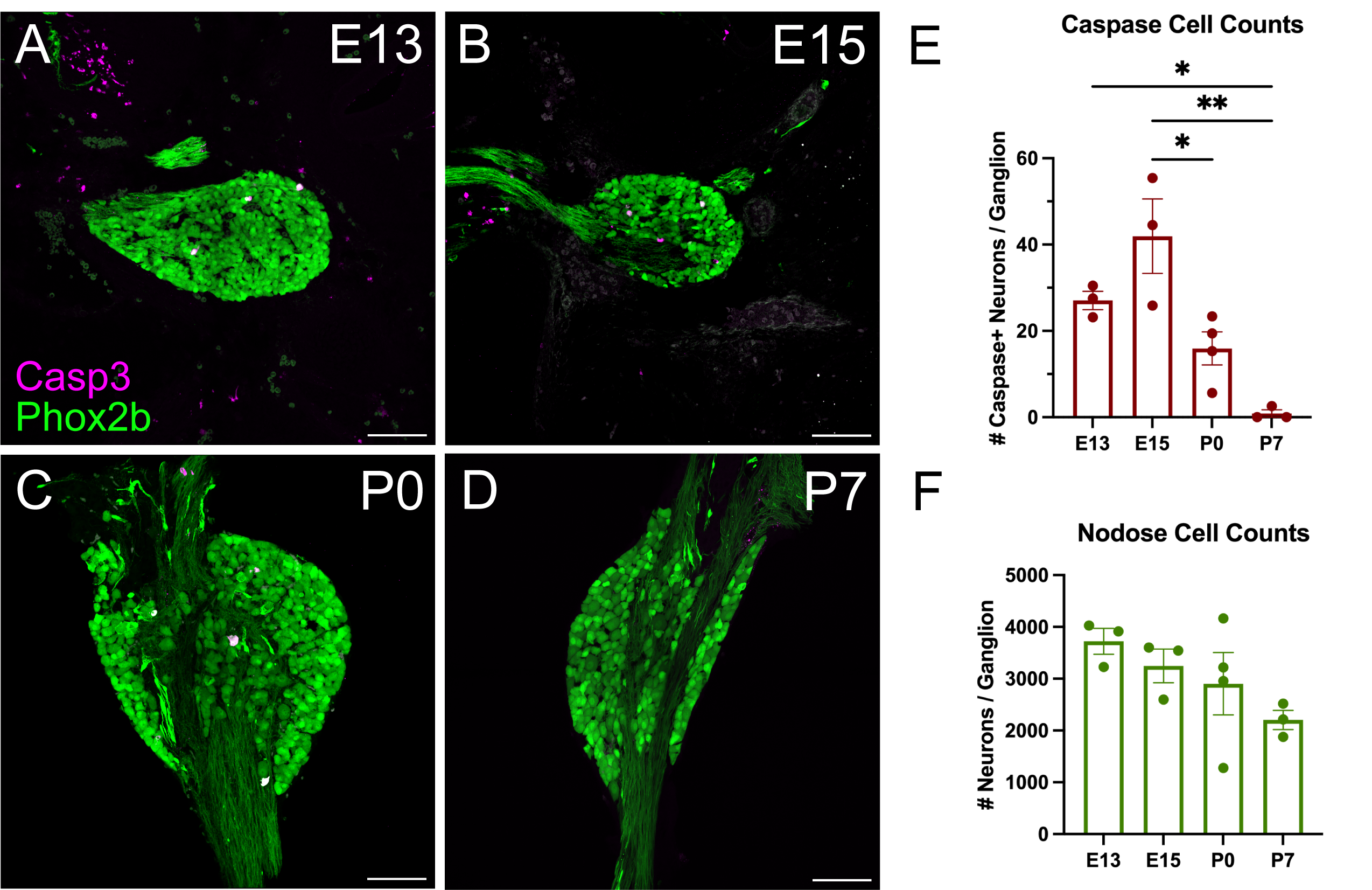

Supplement: Figure 2-1 — Ontogeny of cell death in the nodose ganglion. A–D, Representative images of the right nodose ganglion on E13, E15, P0, and P7 from Phox2bcre;Tomato mice. Immunohistochemistry was used to label the apoptotic marker, activated caspase 3 (magenta), together with endogenous tdTomato labeling (green) of all VSNs. E, Quantification of the number of activated caspase-positive neurons per ganglion on E13, E15, P0, and P7 (n = 3–4 mice/group). *p < 0.05, **p < 0.01 comparing all groups by one-way ANOVA and Tukey’s correction for multiple comparisons. Download Figure 2-1, TIF file. [file enu-eN-NWR-0511-22-s03.tif]

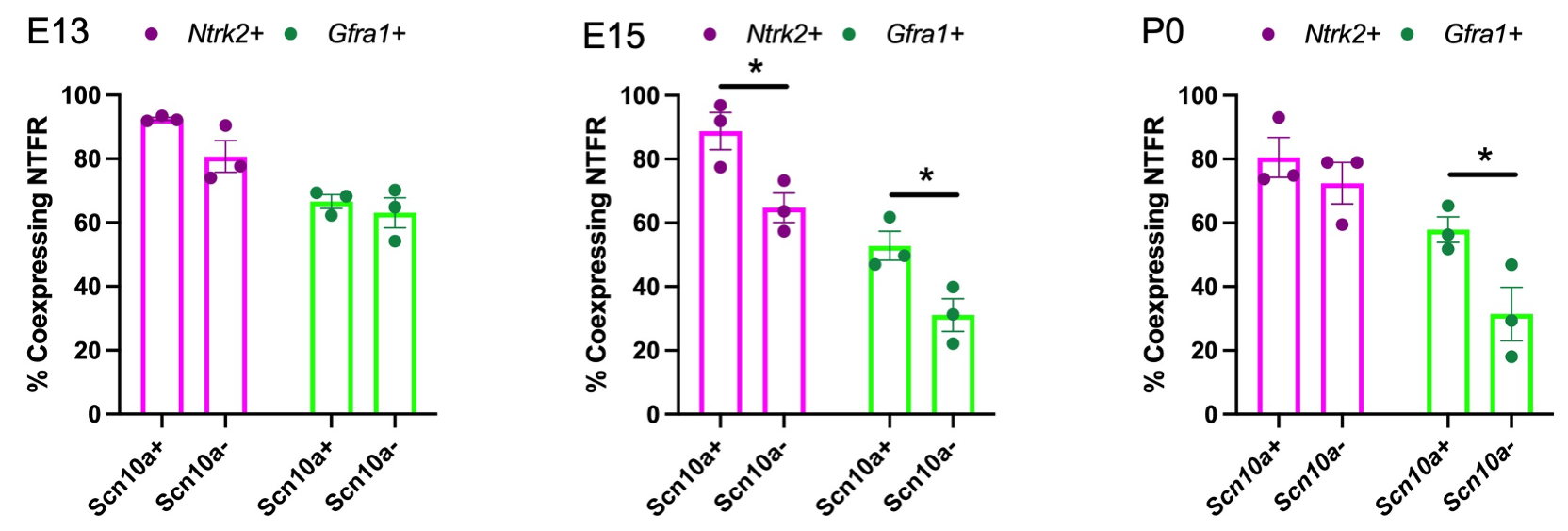

Supplement: Figure 3-1 — Neurotrophic factor receptor expression in Scn10a+ and Scn10a– neurons across development. The percentage of neurons coexpressing either Ntrk2 or Gfra1 was measured on E13, E15, and P0 (n = 3 mice/group). *p < 0.05 as analyzed by two-sided t-test at each age. Statistics for Ntrk2 and Gfra1 were performed separately, then graphed together. Download Figure 3-1, TIF file. [file enu-eN-NWR-0511-22-s04.tif]

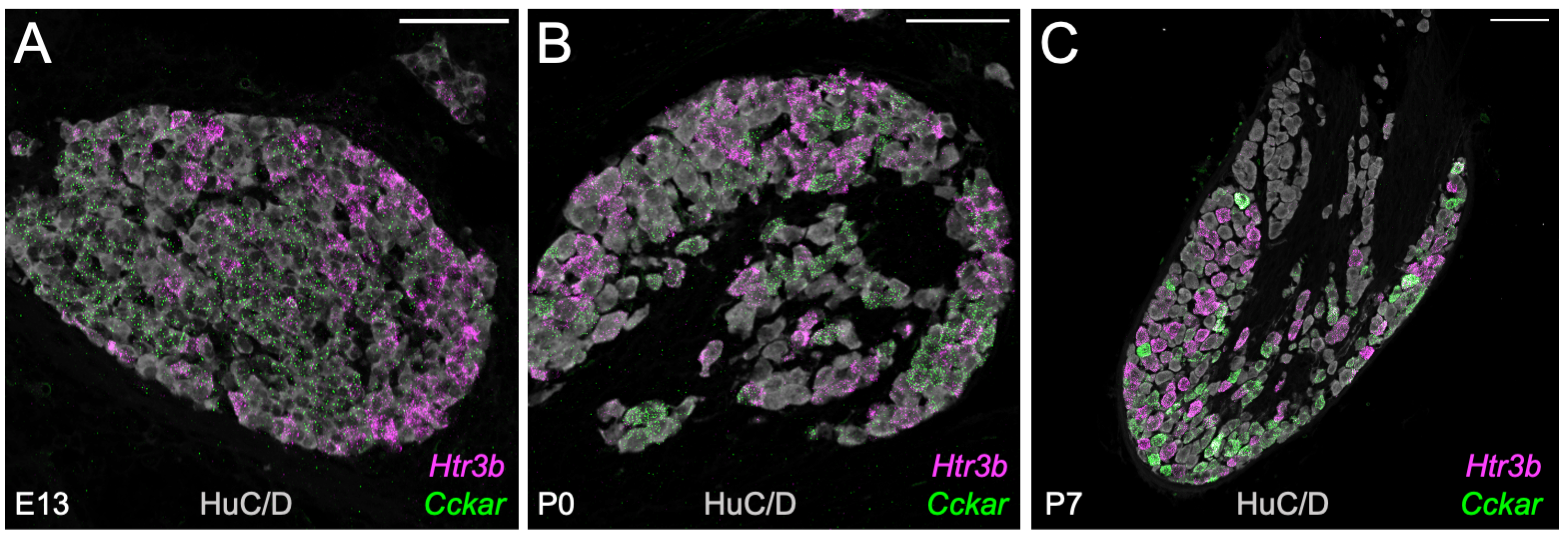

Supplement: Figure 4-1 — Early specification of subpopulations of vagal neurons expressing Htr3b. A–C, Representative images of the right nodose ganglion on E13, P0, and P7 labeled for Htr3b (magenta) and Cckar (green) transcripts and HuC/D. Scale bar, 100 μm. Download Figure 4-1, TIF file. [file enu-eN-NWR-0511-22-s05.tif]
